# miR-638 regulates gene expression networks associated with emphysematous lung destruction

**DOI:** 10.1186/gm519

**Published:** 2013-12-31

**Authors:** Stephanie A Christenson, Corry-Anke Brandsma, Joshua D Campbell, Darryl A Knight, Dmitri V Pechkovsky, James C Hogg, Wim Timens, Dirkje S Postma, Marc Lenburg, Avrum Spira

**Affiliations:** 1 Division of Computational Biomedicine, Department of Medicine, Boston University School of Medicine, 72 East Concord Street Boston, MA 02118, USA; 2 Department of Pulmonary and Critical Care Medicine, University of California, San Francisco, 513 Parnassus Ave, San Francisco, CA 94143, USA; 3 Department of Pathology and Medical Biology, University Medical Center Groningen, University of Groningen, Hanzeplein 1, 9713 Groningen, Netherlands; 4 University of Groningen, University Medical Center Groningen, Groningen Research Institute for Asthma and COPD (GRIAC), Hanzeplein 1, 9713 Groningen, Netherlands; 5 Bioinformatics Program, Boston University, 44 Cummington Street Boston, MA 02215, USA; 6 UBC James Hogg Research Centre, Institute for Heart and Lung Health, St Paul’s Hospital and Department of Pathology and Laboratory Medicine, University of British Columbia, 1081 Burrard St Vancouver, BC V6Z 1Y6, Canada; 7 School of Biomedical Sciences and Pharmacy, University of Newcastle, University Drive Callaghan, New South Wales 2308, Australia; 8 Respiratory Division, Department of Medicine, University of British Columbia, The Jack Bell Research Center, 2660 Oak Street Vancouver, BC V6H 3Z6, Canada; 9 Department of Pulmonary Diseases, University of Groningen, University Medical Center Groningen, Hanzeplein 1, 9713 Groningen, Netherlands

## Abstract

**Background:**

Chronic obstructive pulmonary disease (COPD) is a heterogeneous disease characterized by varying degrees of emphysematous lung destruction and small airway disease, each with distinct effects on clinical outcomes. There is little known about how microRNAs contribute specifically to the emphysema phenotype. We examined how genome-wide microRNA expression is altered with regional emphysema severity and how these microRNAs regulate disease-associated gene expression networks.

**Methods:**

We profiled microRNAs in different regions of the lung with varying degrees of emphysema from 6 smokers with COPD and 2 controls (8 regions × 8 lungs = 64 samples). Regional emphysema severity was quantified by mean linear intercept. Whole genome microRNA and gene expression data were integrated in the same samples to build co-expression networks. Candidate microRNAs were perturbed in human lung fibroblasts in order to validate these networks.

**Results:**

The expression levels of 63 microRNAs (*P* < 0.05) were altered with regional emphysema. A subset, including miR-638, miR-30c, and miR-181d, had expression levels that were associated with those of their predicted mRNA targets. Genes correlated with these microRNAs were enriched in pathways associated with emphysema pathophysiology (for example, oxidative stress and accelerated aging). Inhibition of miR-638 expression in lung fibroblasts led to modulation of these same emphysema-related pathways. Gene targets of miR-638 in these pathways were amongst those negatively correlated with miR-638 expression in emphysema.

**Conclusions:**

Our findings demonstrate that microRNAs are altered with regional emphysema severity and modulate disease-associated gene expression networks. Furthermore, miR-638 may regulate gene expression pathways related to the oxidative stress response and aging in emphysematous lung tissue and lung fibroblasts.

## Background

Chronic obstructive pulmonary disease (COPD) is a growing epidemic and currently the third leading cause of death in the US [1]. Mechanisms leading to the development of COPD are poorly understood, and there are currently no effective therapies that modify long-term lung function decline in patients with the disease.

The heterogeneity of COPD (that is, patients affected by varying degrees of emphysema and small airway disease) may account for some of the difficulty in identifying effective therapies [2]. Although COPD is diagnosed as airflow obstruction that is not fully reversible, measurements of airflow obstruction are weakly correlated with emphysema severity and provide no information on regional differences in emphysema within the lung [3,4]. Emphysema is independently associated with poor clinical outcomes and there is evidence of an emphysema-predominant COPD phenotype in which airflow limitation is less important [5]. Understanding the molecular dysregulation leading to emphysema may lead to a better understanding of this phenotype and more targeted therapeutics.

Prior studies of global gene and microRNA expression in COPD were based on airflow limitation [6-13]. Thus, they were unable to capture the effects of emphysema independent of small airway disease. Furthermore, as these studies were all based on a case–control design, they were unable to account for differences in gene expression associated with varying disease severity within an individual’s lung. We have developed an approach to quantify emphysema severity within different regions of the lung from patients with severe COPD, using micro-computed tomography (micro-CT) data to evaluate mean linear intercept (Lm), a measure of alveolar destruction [14]. Using these Lm measurements, we recently identified a signature of gene expression associated with increasing regional emphysema severity within individual lungs [15]. We found that many of these genes are involved in biological pathways such as inflammation, extracellular matrix (ECM) remodeling, and tissue repair. Furthermore, we were able to predict novel therapeutics using our emphysema gene signature and the Connectivity Map, showing that the compound tripeptide Gly-His-Lys (GHK) reversed the emphysema-associated gene expression changes [16]. However, the molecular mechanisms responsible for the disease-associated changes in gene expression within the lung are not well understood.

MicroRNAs are 19 to 25 nucleotide non-coding RNAs that regulate gene expression through inhibition of mRNA translation or induction of mRNA degradation [17]. It is thought that they fine tune complex regulatory networks by inhibiting multiple genes at once [18,19]. They have repeatedly been implicated in lung disease and are altered with airflow obstruction; however, their role in emphysema is unclear [12,13,20]. MicroRNAs are beginning to be studied in clinical trials as potential biomarkers and therapeutics; thus, understanding how they contribute to disease pathology could lead to significant clinical impact [21].

In this study, we assessed whether microRNA expression changes as regional emphysema severity increases within an individual’s lung and whether these microRNA changes regulate gene expression alterations associated with disease. We performed microRNA profiling in the same lung tissue samples in which we analyzed mRNA expression and evaluated the relationship between microRNA and mRNA expression changes and emphysema severity. A candidate microRNA, miR-638, was further studied in human lung fibroblasts derived from patients with severe COPD to better understand its role in regulating gene expression patterns associated with emphysema pathogenesis *in vivo*.

## Methods

A schematic of the study design is shown in Additional file 1: Figure S1.

### Sample acquisition and preparation

A detailed description of sample preparation and acquisition has been described previously [14,22]. Briefly, lungs were obtained from six subjects undergoing lung transplantation for severe COPD and two donors without COPD. Lungs were cut into 2 cm slices along the axial plane and a cluster of 1 cm cores was taken from each slice. One core from each slice was evaluated by micro-CT while RNA and microRNA were extracted from an adjacent core. Emphysema severity was calculated for each cluster by mean linear intercept (Lm), an estimate of alveolar size, using the micro-CT images. Approximately 1,000 contiguous images were obtained from each scanned core and Lm was measured at 20 regularly spaced intervals in each scan. Lm was calculated by placing a grid of parallel lines over each image and determining the sum of the length of all grid lines divided by the number of intercepts between the alveolar septae and grid lines. Thus, a high Lm corresponds to more severe emphysema as there will be fewer alveolar septae for the grid lines to intercept, and thus a lower denominator.

This study was approved by the institutional review boards at University of British Columbia, Boston University Medical Campus, University of Groningen (for fibroblast work), and University of Pennsylvania (where lungs were procured). The study conforms to the Helsinki Declaration. Written informed consent for use of these specimens and the relevant clinical and radiological data required for this research were obtained from each patient prior to surgery and from the next of kin of the persons whose donated lung was released for research.

### RNA isolation and microarray processing

RNA was extracted from tissue cores and fractionated into high molecular weight (mRNA-containing) and low molecular weight (LMW; microRNA-containing) portions using the miRNeasy mini kit (QIAGEN, Valencia, CA, USA). RNA fraction integrity was assessed using the Agilent 2100 Bioanalyzer and purity was measured using the NanoDrop spectrophotometer.

LMW RNA (<200 ng ng) was processed and hybridized onto NCode version 3 microRNA microarrays (Invitrogen, Carlsbad, CA, USA) as previously described [23]. These arrays contained probes for 1,053 microRNAs from 6 species, including 467 human microRNAs, printed in triplicate. Raw data were extracted using GenePix Pro 4.0 (Axon/Molecular Devices, Sunnyvale, CA, USA).

The mRNA data were generated using Human Exon 1.0 ST microarrays as described in our previously published gene-expression study [15].

### Microarray normalization

Raw microRNA data underwent preprocessing and filtering as previously described [23]. Briefly, the data were quantile normalized, and log transformed (limma package, Bioconductor, R) [24,25]. A microRNA probe was called 'present' if its signal intensity was two standard deviations above the average of the background probes in the sample. MicroRNA probes were filtered out if they were not 'present' in at least 80% of the samples. If two or three replicate probes were called present, the median of the present replicates was taken to obtain a single expression value within each sample. If 0 or 1 probes were called present, the microRNA was filtered out. Samples were then filtered based on two quality metrics: (1) principal component analysis, whereby samples were excluded if they were significant outliers based on the first two principle components; and (2) present/absent filtering, whereby samples were excluded if less than 50% of all human microRNA probesets were 'absent' in the sample. Three samples were excluded by at least one of these two quality metrics.

Transcript-level gene expression values were generated for our previously published gene expression paper via the robust multichip average (RMA) algorithm (affy package, Bioconductor, R) [15,26]. Gene symbols were retrieved using the Entrez Gene Custom chip definition file [27]. Raw data for microRNA and gene expression are publicly available at the Gene Expression Omnibus (GEO) under the accessions GSE49881 and GSE27597, respectively.

### Data analysis

MicroRNA (and gene) expression values were related to emphysema severity using linear mixed-effects models that controlled for differences between patients and position in the lung from which the sample was taken (nlme package, Bioconductor, R) [28]. Two linear mixed effects models were used:

(1) $${\mathrm{miRNA}}_{i}=\beta_{0}+\beta_{\mathrm{Slice}}*\mathrm{Slice}+\beta_{\mathrm{Patient}}*\mathrm{Patient}+\epsilon$$

(2) $$\begin{array}{ll} {\mathrm{miRNA}}_{i}= & \beta_{0}+\beta_{\mathrm{Slice}}*\mathrm{Slice}+\beta_{\mathrm{Lm}}*\mathrm{Lm}\\ & +\beta_{\mathrm{Patient}}*\mathrm{Patient}+\epsilon_{i} \end{array}$$

where miRNA_*i*_ is the response variable representing the log_2_ expression of microRNA *i.* β_Patient_*Patient represents the random effect of 'patient', which controls for samples originating from the same lung. β_Slice_*Slice is a fixed effect that controls for the region in the lung the sample came from (slice number from apex to base). β_0_ is the intercept and ϵ_*i*_ is the error for miRNA_*i*_. In Equation 2 there is a second fixed effect, β_Lm_*Lm, which controls for emphysema severity as defined by the natural log of Lm. miRNA_*i*_ was associated with emphysema severity if the model in Equation 2 (with the emphysema term) fit better than the model in Equation 1 using a likelihood ratio test. Adjustment for multiple comparisons was done by applying the Benjamini-Hochberg false discovery rate (FDR) [29].

The correlation analysis between microRNAs and mRNAs was done using the same equations with genes ('Gene_i_') substituted as the response variable for microRNAs ('miRNA_*i*_'*)* and 'β_miRNA_*miRNA' substituted as the fixed effect 'β_Lm_*Lm' in Equation 2. Thus, this analysis evaluated the correlation between each gene and each microRNA after correction for patient and region of lung. Correlated microRNA-gene pairs (FDR <0.25) were filtered to include only microRNA-predicted target gene pairs as determined by at least one of five target prediction algorithms (Targetscan, Pictar, Tarbase, miRBase, and microRNA.org) [30-34]. The lenient FDR cutoff of 0.25 for significance of microRNA-target gene pairs (corresponding to *P* = 0.002) was used given the hypothesis-generating nature of this correlation analysis. Furthermore, genes were then filtered to include only predicted targets, and targets were validated through *in vitro* experiments.

### Real time PCR validation

Differential expression of eight microRNAs was evaluated with quantitative RT-PCR (qRT-PCR). Three to four samples from two of the eight patients were used to evaluate each microRNA. qRT-PCR was performed using the Taq-Man Small RNA Assay (Applied Biosystems) with 10 ng LMW RNA per manufacturer’s protocol for miR-181d, miR-30c, miR-150, miR18a-3p, miR-211, miR-296-5p, miR-483-3p and miR-638. PCR was carried out using the StepOnePlus Real-Time PCR system with 40 cycles of amplification and data acquisition. Samples were run in triplicate and normalized to U6. Analysis was done using the comparative CT method and expression levels were compared to microarray by correlation analysis.

#### Pathway analysis

Gene set enrichment analysis (GSEA) was used to determine whether pathways that may be biologically important in COPD were associated with microRNAs in our data set [35]. Canonical pathway gene sets were obtained from the Molecular Signature Database (MSigDB) [36]. For each differentially expressed microRNA, a ranked gene list was generated using the *t*-statistics from the linear mixed effect models that correlated mRNA and microRNA expression.

### Isolation, culture, and microRNA transfection in primary lung fibroblasts

Primary lung fibroblasts were cultured from peripheral lung tissue of a subject with stage IV COPD undergoing lung transplant surgery. Cells were isolated and characterized using our explant technique as described previously [37]. Fibroblasts were cultured in complete culture medium (Ham’s F12, 10% fetal bovine serum, penicillin, streptomycin and glutamin (all from Lonza, Verviers, Belgium)) and used for experiments after five passages.

Transfection experiments (n = 3) with miR-638 inhibitors and controls were performed using the HiPerFect transfection reagent (QIAGEN). In short, 2.5 × 10^5^ fibroblasts per well were seeded in six-wells plates. MicroRNA inhibitors in a final concentration of 50 nM (QIAGEN) for mir-638 were added dropwise to the cells. The Inhibitor negative control from QIAGEN was used as the negative control. The transfection reagent was removed after 24 hours and cells were washed with Ham’s culture medium without fetal bovine serum and harvested using trypsin and used for RNA isolation. RNA was isolated using the miRNeasy kit.

RNA was hybridized to Affymetrix Human Gene 1.0 ST arrays. Differential expression between inhibitor and control experiments was determined by empirical Bayes-moderated *t*-tests. Differences in overall expression of miR-638 predicted targets between inhibitor and control experiments was determined by two-tailed Kolmogorov-Smirnov testing (limma, R) [24].

### Pathway analysis in fibroblasts and comparison of fibroblast and emphysema datasets

GSEA analysis was done to determine biological pathways enriched with miR-638 inhibition. Genes were ranked by fold change difference between control and inhibitor and canonical pathway gene sets were run against this ranked list. Our fibroblast dataset was compared to our emphysema dataset by using a gene set created from the 100 most upregulated miR-638 predicted targets (by fold change) between miR-638 inhibitor experiments and control experiments. This gene set was run against a list of genes ranked by their correlation with miR-638.

## Results

### Study population

Eight sample pairs from different lung slices were obtained from six subjects requiring lung transplant for COPD and two organ donors (64 samples total). Detailed characteristics of subjects and lung specimens were described previously and are shown in Additional file 2[15]. Briefly, the six subjects with COPD had a forced expiratory volume in 1 second (FEV_1_) <25% predicted (severe disease). Subjects with COPD had higher and more variable mean linear intercept (Lm) values for their tissue samples than donors except for one subject who had small airways disease without emphysema. All subjects were former smokers except one donor. Four samples were excluded from further analysis: one as an outlier by Lm, the others as outliers by microRNA microarray quality metrics.

### MicroRNA expression is altered with regional emphysema severity

**Figure 1:**
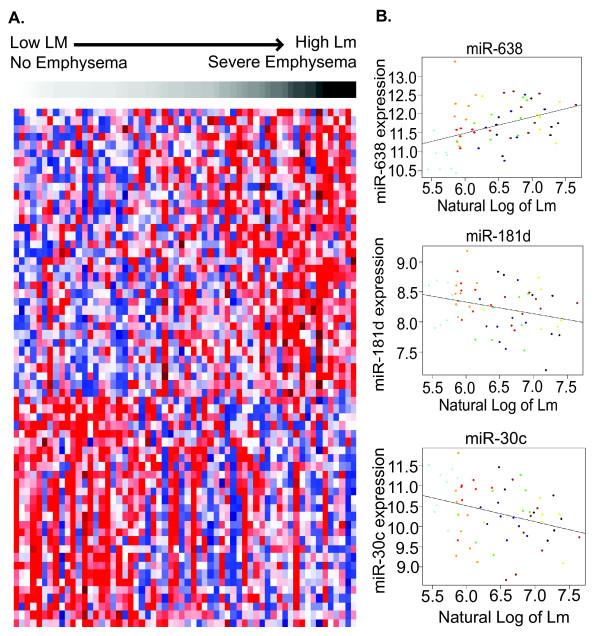
**MicroRNA expression associated with emphysema severity. (A)** Supervised clustering of 63 microRNAs significantly correlated with emphysema progression (*P* < 0.05). Samples are arranged in columns from low Lm (white) to high Lm (black). Red indicates high relative expression, blue indicates low relative expression. **(B)** Scatter plots of expression values plotted against Lm for three microRNAs. Colors indicate different subjects. As shown in this plot, each of the microRNAs is correlated with Lm across all subjects. However, importantly each microRNA is correlated with Lm within patients as well.

After microarray preprocessing, 397 of the initial 467 human microRNAs were detected above background and used for further analysis. Sixty-three microRNAs were correlated with Lm and thus with regional emphysema severity (*P*-value < 0.05, 20 expected by chance at this threshold; Figure 1; Additional file 3). In contrast, only 18 microRNAs were correlated with position in the lung the sample came from (for example, apical or basal, *P* < 0.05), and only three of these 18 microRNAs correlated with Lm (Additional file 4). The microRNAs upregulated with increasing emphysema severity included broadly conserved and poorly conserved microRNAs, with the most upregulated including miR-520e and miR-302d from the miR-93 family, miR-92a, miR-638, miR-211, and miR-150. The downregulated microRNAs included clusters from several broadly conserved families. These include Let-7c, Let-7d, Let-7e, and Let-7f from the Let-7/miR-98 family, miR-181c and miR-181d from the miR-181/4262 family, and miR-30a-3p, miR-30c, miR-30e-5p, and miR-30e-3p from the miR-30/384-5p family. The association with regional emphysema severity was validated using qRT-PCR. Expression values derived from microarray and RT-PCR data were significantly correlated for four of the eight miRNAs (Pearson; *P* ≤ 0.05; Additional file 1: Figure S2) and just below statistical significance but trending in the correct direction for a fifth microRNA, miR-181d (*P* = 0.06). The expression of these five microRNAs and two of the other three all trended in the expected direction (same as in the array data) with increasing Lm.

### Gene expression is correlated with microRNA expression in the emphysematous lung

To identify which microRNAs may regulate gene expression associated with emphysema pathogenesis, we generated a microRNA-mRNA correlation network by integrating the microRNA expression data with global mRNA expression data generated from the same lung specimens. We found that 1,079 microRNA-mRNA pairs were significantly correlated (371 negative correlations, 708 positive correlations) using linear mixed effect models (FDR <0.25) and were predicted to interact by at least one of five sequence-based target prediction algorithms.

**Figure 2:**
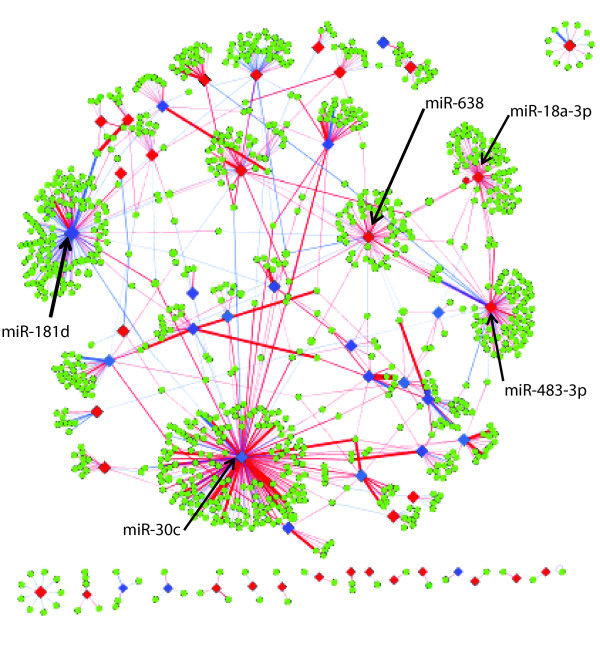
**MicroRNA-mRNA co-expression network for the differentially expressed microRNAs.** Diamonds represent microRNAs and green circles represent target genes. Lines identify significant correlations between microRNA and gene expression patterns (FDR <0.25) with the microRNA predicted to target the gene by at least one of five sequence-based prediction algorithms. Red lines indicate positive correlations and blue lines indicate negative correlations. Red nodes indicate upregulated microRNA expression with increased emphysema severity, blue indicates downregulation. The thickness of the line correlates to the number of algorithms that predict the interaction.

Of the 63 differentially expressed microRNAs, 51 had at least one positively or negatively correlated predicted target at FDR <0.25 (Figure 2; Additional file 5). The most highly connected microRNAs in the network included miR-638, miR-18a-3p, miR-483-3p, miR-181d, and miR-30c, which had greater than 50 positively or negatively correlated predicted targets, suggesting that these microRNAs may be important regulators of gene expression associated with emphysema severity.

### MicroRNAs regulate pathways involved in COPD pathogenesis

**Figure 3:**
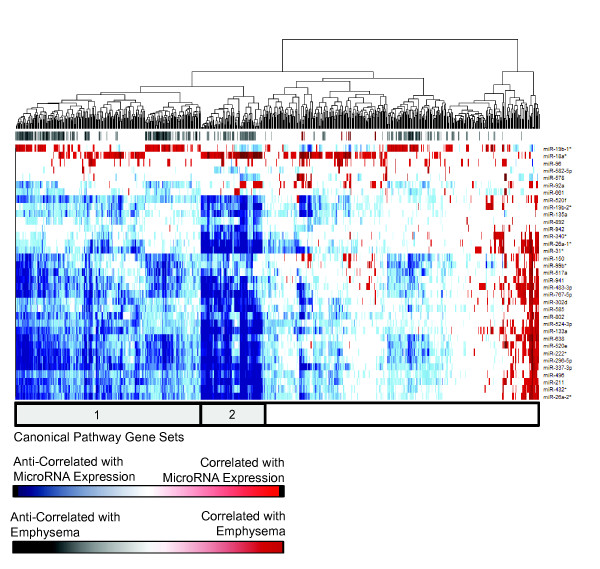
**MicroRNAs are inversely correlated with biological pathways downregulated with increasing regional emphysema severity.** Expression-based association matrix of the 35 significantly upregulated microRNAs (rows) and those MSigDB canonical pathway gene sets (columns) that are significantly enriched amongst genes correlated with the microRNA (FDR <0.25). Red indicates positive correlation, blue indicates negative correlation, and white indicates no significant correlation. The color bar at the top of the heatmap indicates pathways also correlated with emphysema (FDR <0.25) where red indicates positive correlation and grey negative correlation. The grey and white bar below the heatmap highlights clusters of downregulated pathways. The group of pathways indicated by bar 1 includes those most downregulated with emphysema (for example, those involved in ECM maintenance and cell signaling). The second bar highlights an additional cluster of pathways most anti-correlated with upregulated microRNA expression; it includes mainly pathways involved in RNA transcription and processing, cell cycle progression, mitochondrial functioning, DNA repair, and protein degradation.

To gain insight into functional significance of the microRNA-mRNA interactions, we next used GSEA to determine the association between each microRNA and 880 canonical pathway gene sets. Despite the microRNA-mRNA target analysis showing both positive and negative correlations, the significantly enriched canonical pathways were almost all inversely correlated with upregulated microRNAs, suggesting an inhibitory role for microRNAs with increasing disease severity. Unsupervised hierarchical clustering of the pathways significantly enriched amongst upregulated microRNAs identified several distinct clusters of downregulated functional categories associated with expression of multiple upregulated microRNAs, including miR-638 (Figure 3). Pathway gene sets most downregulated in emphysema, including those involved in cell surface signaling and ECM maintenance (for example, transforming growth factor β, integrin, and wnt-signaling), were inversely correlated with the expression of multiple upregulated microRNAs. We also identified additional pathways important to emphysema pathology that were inversely correlated with upregulated microRNAs, including pathways in tissue and cell repair (for example, DNA repair and synthesis, cell cycle maintenance and traverse, RNA processing, and proteasome maintenance/ubiquitination), and mitochondrial biogenesis (for example, oxidative phosphorylation, electron transport chain).

### Inhibition of miR-638 in lung fibroblasts leads to overexpression of its predicted targets

**Figure 4:**
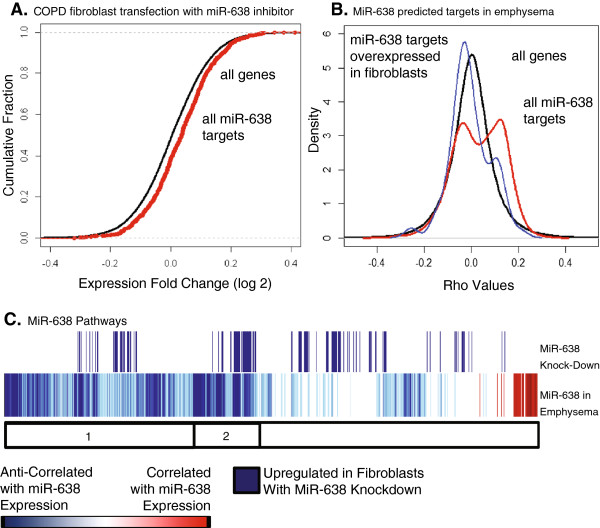
**Changes in miR-638 predicted target gene expression with mir-638 knockdown. (A)** ECDF, (empirical cumulative distribution function) plot showing the *t*-statistics from empirical bayes-moderated *t*-tests for miR-638 predicted target genes (red) versus all genes (black). The predicted targets are shifted right from control in the inhibitor transfected samples, indicating the expected upregulation of target gene expression. Expression of the predicted targets are significantly different between controls and inhibitors by two-sided Kolmogorov-Smirnov tests (*P*-value = 2.576e-10). **(B)** Density plots of the *t*-statistics from correlations between miR-638 expression and gene expression in emphysematous lung tissue for: (1) all genes (black), (2) all miR-638 predicted targets (red), and (3) those miR-638 predicted targets over-expressed in fibroblasts with miR-638 knock-down (blue). The majority of miR-638 correlations are in the positive direction, consistent with the red line shifted right, while the targets over-expressed with miR-638 inhibition are mostly anti-correlated, consistent with the blue line shifted left. **(C)** A magnified view of the miR-638 row extracted from the expression matrix in Figure 4 shows the pathways positively and negatively correlated with miR-638 expression (red and blue, respectively). Pathways upregulated with miR-638 knock-down in fibroblasts are shown above this in blue. This illustrates that many of the pathways overlap, particularly in the sets of pathways associated with cell cycle progression, RNA transcription, and mitochondrial biogenesis (bar 1), and DNA repair (bar 2).

We next examined the specific effects of miR-638 on the expression of its predicted targets in primary human lung fibroblasts obtained from a subject with severe COPD, as fibroblasts play a key role in the aberrant tissue repair and ECM maintenance observed in emphysema pathogenesis [37,38]. miR-638 was chosen as it had many correlated targets, was anti-correlated with multiple pathways involved in ECM dysregulation and tissue repair by GSEA, and has been shown previously to be expressed in normal human lung fibroblasts [39]. We knocked down miR-638 in primary human lung fibroblasts and measured global gene expression using Affymetrix Human Gene 1.0 ST arrays. We observed a significant enrichment of miR-638 predicted targets amongst upregulated genes in miR-638 knockdown experiments compared to controls (*P* = 2.6 × 10^-10^, Kolmogorov-Smirnov test; Figure 4A). miR-638 inhibition caused modest changes in the expression of many target genes (1.15 to 1.4 fold change for significant genes in our experiments), similar to previously published studies on microRNA transfection [40,41].

### miR-638 targets are involved in oxidative stress-related pathways and are anti-correlated with miR-638 expression in emphysematous lung tissue

To evaluate the functional significance of miR-638 expression in emphysema, we identified the canonical pathways affected by miR-638 knock-down in fibroblasts, and studied the overlap with genes and pathways correlated with miR-638 in emphysematous lung tissue. GSEA analysis revealed enrichment of many of the same biological pathways among the genes increased in fibroblasts after miR-638 inhibition as those whose expression was anti-correlated with miR-638 expression in emphysema (FDR <0.25; Figure 4C; Additional file 6: Table S5). miR-638 inhibition in fibroblasts led to upregulation of pathway gene sets involved in DNA repair, cell-cycle maintenance and traverse, RNA transcription, mitochondrial biogenesis, telomere maintenance, and processing of damaged proteins. Sixty-nine (56%) of these pathways were also anti-correlated with miR-638 expression in emphysematous lung tissue, and previously implicated in oxidative stress and accelerated lung aging responses.

The 100 most upregulated predicted targets by fold change with miR-638 inhibition in fibroblasts were enriched amongst predicted targets anti-correlated with miR-638 expression in emphysema (*P* < 0.001, Kolmogorov-Smirnov test; Figure 4B). These predicted targets included genes involved in cell proliferation (*ADAM15*, *ARHGDIA*, *COMMD1*, *DHCR7*, *HDAC5*, *MAD2L2*, *PFKL*, *YPEL3*), autophagy and protein degradation (*ATG9A*, *GANAB*), mitochondrial functioning (*DHCR7*, *ERAL1*, *SLC25A1*, *STARD3*, *TOMM40*), DNA damage response (*APBB1*, *KRT7*, *MAD2L2*), oxidative stress response (*CARHSP1*), and ECM remodeling (*LTBP4*). These results demonstrate that by modulating miR-638 in lung fibroblasts obtained from a patient with COPD, we can reproduce emphysema-related patterns of gene expression observed in lung tissue and further suggest a role for miR-638 in emphysema pathogenesis.

## Discussion

In this study, we have shown that regional emphysema severity is associated with alterations in microRNA expression and that a subset of these microRNA changes are correlated with changes in their predicted gene target expression. While 51 of the 63 altered microRNAs correlated with at least one of their predicted targets, a much smaller subset had many correlated targets. Five of these microRNAs (miR-638, miR-181d, miR-18a-3p, miR-30c, and miR-483-3p) were correlated with ≥50 of their predicted targets, suggesting that these microRNAs may play a key role in gene regulation in emphysema.

Unique strengths of our study include our ability to relate microRNA changes to the emphysematous component of COPD, to account for regional differences in emphysema within an individual patient’s lung, and to relate microRNA changes to mRNA changes within these same samples. Other studies of microRNA expression in COPD have used airflow obstruction alone as the COPD phenotype to associate with molecular alterations [12,13]. However, emphysema correlates poorly with severity of airflow obstruction and contributes independently to outcomes in COPD [2,4,42]. In addition, emphysematous destruction and small airway disease occur to different degrees throughout the lungs of patients with COPD, and airflow obstruction measurements cannot capture these differences. We isolated multiple samples from regions within an individual patient’s lung varying by degree of emphysema and by location (for example, apical, basal), and used Lm to quantify the degree of emphysema severity within each sample. By using this study design, we were able to specifically identify those microRNAs whose expression is related to emphysema severity/progression within the lung, controlling both for microRNA expression differences between individuals and for differences in lung location the sample came from (for example, apical, basal). Furthermore, the unique study design allowed us to identify microRNA-mRNA networks that underlie the pathogenesis of emphysematous destruction within an individual’s lung.

An analysis of microRNA-target interactions revealed numerous significant negative and positive interactions suggesting that microRNAs are an important part of the regulatory network in emphysema. Somewhat surprisingly, 66% of significant correlations were in the positive direction. Although this seems counterintuitive, given that microRNAs inhibit gene expression, positive correlations are noted throughout the literature, including in a study of human airway microRNA and gene expression [19,43-45]. Positive correlations are attributed to microRNAs acting together in a network with other regulatory processes to influence gene expression (Additional file 1: Figure S3). Both microRNAs and the mRNAs they directly inhibit may be under the control of common upstream regulators. This can lead to positive correlation between the expression of the microRNA and mRNA pairs even though the microRNAs are functioning to decrease mRNA expression levels in this context (a coexpressed or 'incoherent' circuit). Negative microRNA-target gene correlations suggest upregulated microRNAs act with other processes to lower target gene expression (an expected or 'coherent' circuit).

Our pathway analysis suggests microRNA-gene interactions may contribute to emphysema pathogenesis by influencing pathways involved in ECM maintenance, the oxidative stress response and lung aging. The pathway gene sets most downregulated with increasing emphysema severity, including those involved in ECM maintenance, were anti-correlated with upregulated microRNA expression. In addition, pathways involved in tissue repair processes (for example, DNA repair, cell cycle traverse, and lysosome maintenance) and mitochondrial functioning, which are dysregulated in accelerating lung aging processes and critical to emphysema pathogenesis, were also significantly enriched amongst genes anti-correlated with upregulated microRNAs. Multiple microRNAs have previously been found to regulate tissue repair processes and to act cooperatively or redundantly to control these functions [46]. Our findings support this and suggest that dysregulation of multiple microRNAs may contribute to emphysema pathogenesis.

We studied the function of miR-638, a microRNA whose expression increases with increasing emphysema severity, in regulating gene expression in human lung fibroblasts. Peripheral lung fibroblasts were studied as they have been implicated in COPD as key regulators of accelerated lung aging and dysregulated ECM repair. Our analysis suggests that the microRNAs changing with emphysema severity may be regulators of these processes. Fibroblasts are the main producers of the ECM and have been shown by us and others to be deficient in collagen contraction, a model property of the repair process, in patients with COPD [37,38]. Accelerated aging processes (for example, cellular senescence) are upregulated in fibroblasts in COPD and have been implicated as mediators of fibroblast functional insufficiency, and thus their decreased contribution to ECM repair, in COPD [47]. miR-638 was chosen as it has many correlated targets and was previously found to be associated with cellular senescence and DNA damage [39,48], suggesting it contributes to accelerated lung aging as well. It is expressed in human lung fibroblasts and its expression increases as a function of cellular senescence [39]. miR-638 overexpression in bronchial epithelial cells also increases DNA damage and decreases DNA repair, both of which contribute to cellular senescence and tissue repair dysregulation, after exposure to benzo(a)pyrene, a polycyclic aromatic hydrocarbon and component of cigarette smoke [48].

We found that miR-638 knock-down in COPD fibroblasts preferentially leads to over-expression of its predicted targets and that overexpressed targets are enriched amongst gene targets anti-correlated with miR-638 expression in emphysematous lung tissue. Thus, although miR-638 is positively correlated with two-thirds of its predicted targets in emphysema, those targets that are most affected with modulation of this microRNA in COPD fibroblasts are anti-correlated with miR-638 expression in emphysema. While the positive correlations imply that miR-638 is part of a regulatory network in which upstream regulators lead to microRNA-mRNA target coexpression (incoherent regulatory circuit), we show evidence that miR-638 is an important regulator in the network as well, as it is anti-correlated with those targets it directly inhibits most in COPD fibroblasts. These findings clearly suggest that miR-638 contributes to the gene expression changes related to emphysema severity.

**Figure 5:**
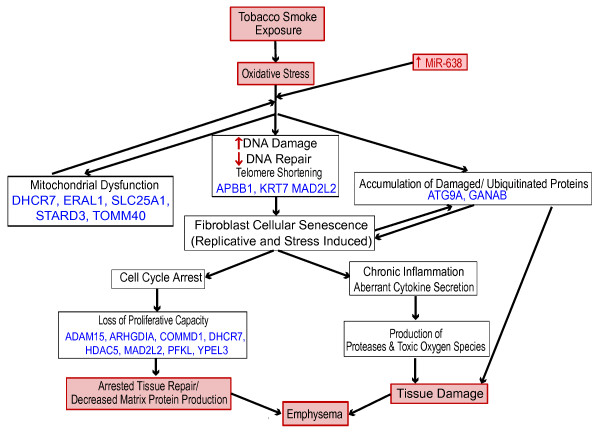
**Potential role for miR-638 in the pathogenesis of emphysema.** GSEA identified multiple pathways dysregulated in the aging response to oxidative stress that were enriched both in fibroblasts after miR-638 inhibition and anti-correlated with miR-638 expression in emphysema. The oxidative stress response to chronic tobacco smoke exposure leads to: (1) DNA damage and telomere shortening that stimulates cellular senescence in fibroblasts - senescent fibroblasts can no longer proliferate, and thus cannot effectively repair damaged ECM, and senescent cells also stimulate chronic inflammation leading to increased proteolysis and tissue damage; (2) mitochondrial dysfunction, leading to further production of reactive oxygen species and chronic oxidative stress; and (3) accumulation of degraded proteins and organelles with dysregulation in removal processes (autophagy and proteosomal), leading to further tissue damage and cellular senescence. We propose that miR-638 is involved in fine-tuning these processes and as its expression is increased with emphysema it contributes to increased cellular senescence, decreased tissue repair and increased tissue damage. Enriched pathways were found in all of these dysregulated processes. Predicted miR-638 targets that participate in these functions and are over-expressed with miR-638 knock-down and anti-correlated with miR-638 in emphysema are listed in blue.

miR-638 inhibition in fibroblasts led to upregulation of pathways that were also dysregulated in emphysematous lung tissue and involved in responses consistent with accelerated lung aging and the oxidative stress response. Furthermore, several miR-638 gene targets involved in these processes were dysregulated *in vitro* with miR-638 inhibition and anti-correlated with miR-638 expression in lung tissue with increasing emphysema severity. Accelerated aging, which can occur independently of and earlier than chronological aging, is a collection of molecular and cellular alterations that has been implicated in several diseases (for example, emphysema, Parkinson’s disease, diabetes, coronary artery disease). Some of these processes include: (1) cellular senescence, telomere attrition, persistent DNA damage response, and cell cycle arrest, leading to decreased cell proliferation and stimulation of T-helper type 1-associated inflammation; (2) mitochondrial dysfunction leading to increased production of free radicals and oxidative stress; and (3) decreased removal of damaged proteins leading to further cellular senescence and increased tissue damage [49-52]. Chronic exposure to cigarette smoke leads to chronic oxidative stress that has been implicated in turning on these aging processes prematurely. This is hypothesized to lead to the tissue destruction and inability to replace cells that we observe in emphysema. Given our pathway and microRNA-target interaction analyses, we propose that miR-638 contributes to accelerated lung aging and oxidative stress responses in emphysema (Figure 5).

While most studies of microRNAs in the lung show regulation of just one or a few genes, a strength of our study is that we are able to show that microRNAs can alter the expression of multiple genes leading to pathology. Amongst these genes is *HDAC5*, which encodes histone deacetylase 5, and has been shown to decrease with increasing COPD severity. Through epigenetic modification, this decrease in HDAC5 has been hypothesized to contribute to cell cycle arrest and increased inflammation [35]. Another of these genes, *ADAM15*, which encodes one of the disintegrin and metalloproteinase domain-containing proteins, has been shown to be protective against emphysema as *ADAM15*^−/−^ mice develop more severe emphysema in response to cigarette smoke [53]. Many of the other gene targets have never been studied in COPD, and thus represent an area for future investigation as possible important regulators of disease.

While only a relatively small number of patients were profiled in this study, we obtained multiple samples per patient, allowing us to examine the microRNA alterations associated with regional differences in emphysema severity. Sequence-based computational algorithms for predicting microRNA-mRNA interactions generally have poor sensitivity and specificity. However, by including predictions from multiple algorithms and by intersecting this with microRNA-gene coexpression networks generated in subjects with severe emphysema we were able to identify a microRNA capable of modulating its targets in emphysema-related pathways *in vitro*.

Using our analytical approach we have predicted which microRNAs are most important to emphysema pathogenesis and how they are interacting with target genes to contribute to disease. Additional studies are needed to investigate how the microRNAs specifically contribute to the emphysema phenotype. In addition, given the vast public health impact of COPD, the scarcity of treatment options, and the recent advances in the development of microRNA-directed therapeutics, future work may focus on these microRNAs as potential treatment targets in emphysema. We have shown that the downstream inhibition of multiple genes by at least one of these microRNAs, miR-638, likely functions to affect emphysema pathogenesis. Thus, targeting microRNAs in emphysema may have a significant impact by altering the expression of many genes relevant to disease pathogenesis.

## Conclusions

This study demonstrates that microRNA expression is altered with regional emphysema severity and that these microRNAs can regulate the gene expression changes associated with emphysema. Our study design allowed us to examine emphysematous destruction independent of airflow limitation and to control for heterogeneity of emphysema severity between and within patients, which limited previous analyses of microRNA expression in COPD. We identified several microRNAs that may be involved in emphysema pathogenesis and appear to modulate disease-specific gene expression networks. We demonstrated that miR-638, which was upregulated in emphysema, likely participates in COPD pathology by responding to oxidative stress, contributing to an accelerated lung aging response and leading to an impaired ability of the damaged lung to replace the damaged ECM. MicroRNAs identified as important to emphysema pathogenesis are intriguing candidates for further study in biomarker and therapeutic target development in emphysema.

## Abbreviations

COPD: chronic obstructive pulmonary disease; CT: computed tomography; ECM: extracellular matrix; FDR: false discovery rate; GSEA: gene set enrichment analysis; Lm: mean linear intercept; LMW: low molecular weight; MSigDB: Molecular Signature Database; qRT-PCR: quantitative real time polymerase chain reaction.

## Competing interests

The authors declare that they have no competing interests.

## Authors’ contributions

Stephanie A Christenson and Corry-Anke Brandsma contributed as co-first authors. AS, SAC, CAB, JDC, DAK, DSP, ML, and WT conceived and designed the experiments. SC analyzed the array data with the assistance of AS, JC, and ML. CB and DVP performed the *in vitro* experiments. JCH, CAB, DSP, and WT contributed materials. SAC, CAB, JDC, MEL, JCH, DSP, WT and AS wrote and/or revised the manuscript. All authors read and approved the final version for publication.

## Supplementary Material

Additional file 1: Figure S1 Overview of the study design. **Figure S2.** validation of microRNA alterations in emphysema by RT-PCR. **Figure S3.** microRNA-gene network interactions. Click here for file

Additional file 2: Table S1 Subject demographics for the eight participants, six requiring transplantation for COPD and two organ donors. Those with COPD had a forced expiratory volume in 1 second / forced vital capacity (FEV_1_/FVC) <70% predicted and FEV_1_ <25% predicted. One COPD subject had small airway disease only (no emphysema). Some subjects carried other diagnoses: one COPD subject had von Willebrand disease, one COPD subject and one donor had hypertension, and one COPD subject had α1 antitrypsin disease. All subjects were former smokers with the exception of one donor who was a never-smoker. Click here for file

Additional file 3: Table S2 MicroRNAs differentially expressed in emphysema, statistics. Lm coefficient (log 2 fold change, 'Beta'), *P*-value, and *t*-statistic for the differentially expressed microRNAs. Click here for file

Additional file 4: Table S3 MicroRNAs differentially expressed by location in the lung, statistics. Lm coefficient (log 2 fold change, 'Beta'), *P*-value, and *t*-statistic for the differentially expressed microRNAs. Click here for file

Additional file 5: Table S4 Number of correlated and anti-correlated targets for differentially expressed microRNAs. Click here for file

Additional file 6: Table S5 Pathway gene sets anti-correlated with miR-638 in emphysema and upregulated with miR-638 knock-down in fibroblasts. Normalized enrichment score (NES), *P*-value, and FDR value for GSEA of all overlapping gene sets in both datasets at FDR <0.25. Gene sets are sorted by function, all associated with the oxidative stress and abnormal lung aging response: protein processing, mitochondrial dysfunction, DNA damage response, carbohydrate metabolism, diseases associated with accelerated aging, cell cycle traverse, and RNA transcription and processing. Click here for file

## Support

This work was funded by the National Heart, Lung and Blood Institute (R01HL095388 to AS and MEL), National Center for Advancing Translational Science (UO1 TR000157 to AS and MEL), British Columbia Lung Association, Canadian Institute for Health Research (to JH and DAK), and the Dutch Asthma Foundation and European Respiratory Society (International Research Fellowships to CAB) and the ‘Stichting Astma Bestrijding’(to CAB), National Science Foundation (Integrative Graduate Education and Research Traineeship to JDC).
