# Supplementary material for: miR-638 regulates gene expression networks associated with emphysematous lung destruction
Source: Genome Med. 2013 Dec 31;5(12):114. doi: 10.1186/gm519 (PMC3971345; doi:10.1186/gm519)
Supplement: Additional file 1: Figure S1 — Overview of the study design. Figure S2. validation of microRNA alterations in emphysema by RT-PCR. Figure S3. microRNA-gene network interactions. [file gm519-S1.pdf]

Additional file 1

Figure S1. Overview of the study design. Frozen explanted lungs were cut into 2cm slices along the axial plan. One cm cores were taken from each 2 cm slice. 1 core from each slice was evaluated by micro-CT. RNA and microRNA was isolated from an adjacent core. Micro-CT images were used to calculate mean linear intercept (Lm). Micro-CT images from normal lung (left) and emphysematous lung (right) are shown. A grid of parallel lines (shown in red) was placed over each image and Lm was calculated by adding the length of all grid lines and dividing by the number of intercepts between the alveolar septae and the grid lines. RNA and microRNA were hybridized to microarrays and expression was related to Lm using mixed effect linear models. Significant mRNA and microRNA correlations were determined using mixed effect linear models and intersected with target prediction databases to create a microRNA-mRNA target network. GSEA was used to determine which canonical pathways were enriched with emphysema severity and with microRNA differential expression. MiR-638 knock-down in COPD fibroblasts was done for *in vitro* validation. Over-expressed miR-638 targets in these experiments were evaluated in the emphysema dataset as were pathways enriched amongst the over-expressed genes.

Figure S2. Validation of microRNA alterations in emphysema by RT-PCR. Shows the delta-delta CT values for a low LM (less emphysema) and high LM (more emphysema) sample for 8 microRNAs in 2 subjects with emphysema. While all but miR-483-3p were significantly correlated with LM in at least one subject, 4 of the 8 (miR-30c, miR-18a-3p, miR-638, and miR-150) were significantly correlated with the array data by Pearson correlation and one, miR-181d, almost reached statistical significance (values reported in figure).

Figure S3. MicroRNA-gene network interactions. (A) microRNA- target gene correlation can fall anywhere between highly positively correlated and highly anti-correlated. The functions of different interactions are likely to be different based on where they fall in this spectrum, with most interactions leading to fine tuning, but highly anti-correlated microRNA-target interactions indicating an “on-off” function for the microRNA. COPD fibroblast targets are mostly anti-correlated with miR-638 expression in emphysema (coherent) but most of these targets are not highly anti-correlatied, suggesting a tuning function. (B), (C) show schematics of incoherent and coherent circuits.

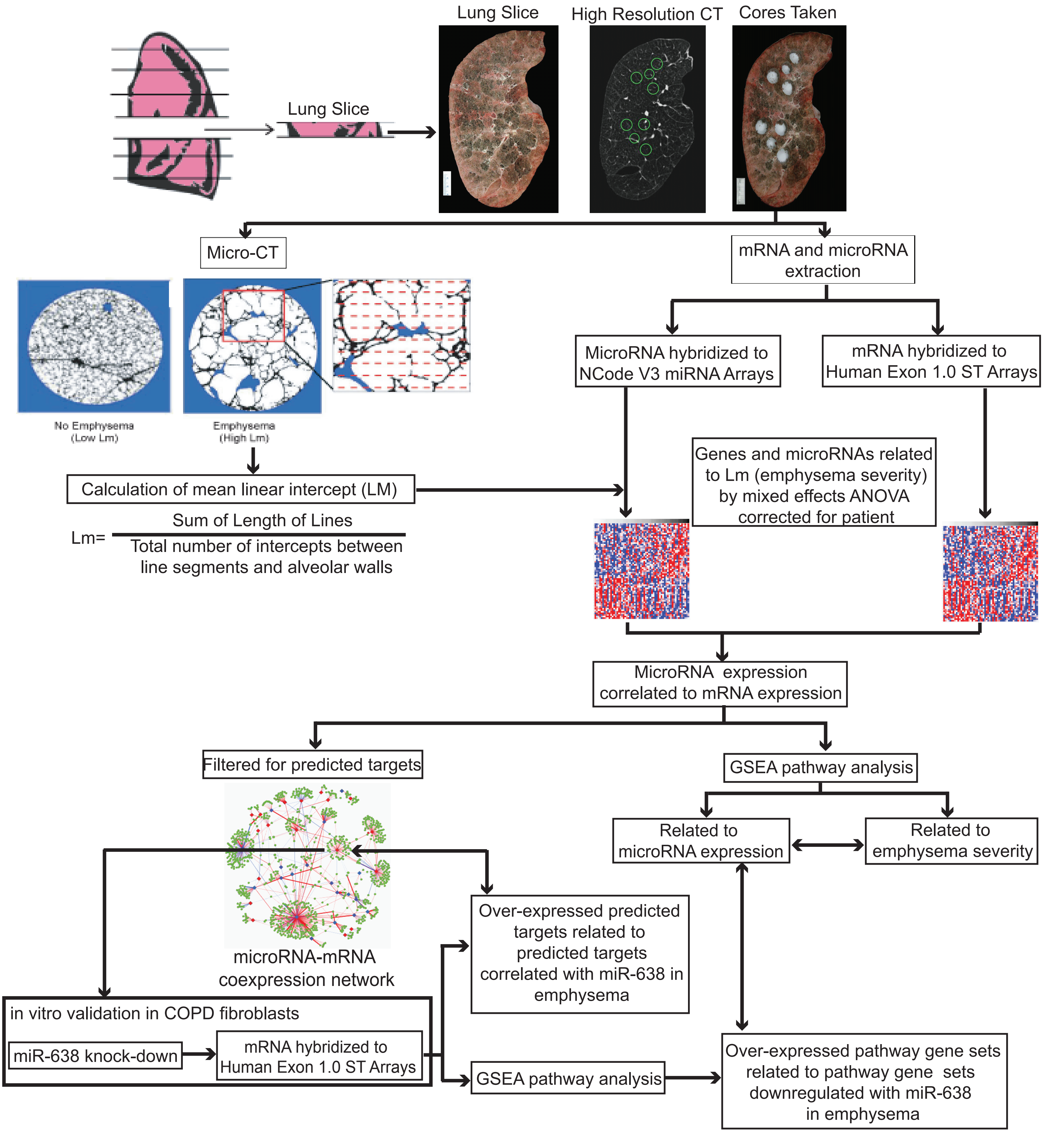
Lung Slice
High Resolution CT
Cores Taken
Lung Slice
Micro-CT
mRNA and microRNA extraction
No Emphysema (Low Lm)
Emphysema (High Lm)
MicroRNA hybridized to NCode V3 miRNA Arrays
mRNA hybridized to Human Exon 1.0 ST Arrays
Genes and microRNAs related to Lm (emphysema severity) by mixed effects ANOVA corrected for patient
Calculation of mean linear intercept (LM)
Lm= Sum of Length of Lines / Total number of intercepts between line segments and alveolar walls
MicroRNA expression correlated to mRNA expression
Filtered for predicted targets
GSEA pathway analysis
Related to microRNA expression
Related to emphysema severity
microRNA-mRNA coexpression network
Over-expressed predicted targets related to predicted targets correlated with miR-638 in emphysema
in vitro validation in COPD fibroblasts
miR-638 knock-down
mRNA hybridized to Human Exon 1.0 ST Arrays
GSEA pathway analysis
Over-expressed pathway gene sets related to pathway gene sets downregulated with miR-638 in emphysema

Figure S1.

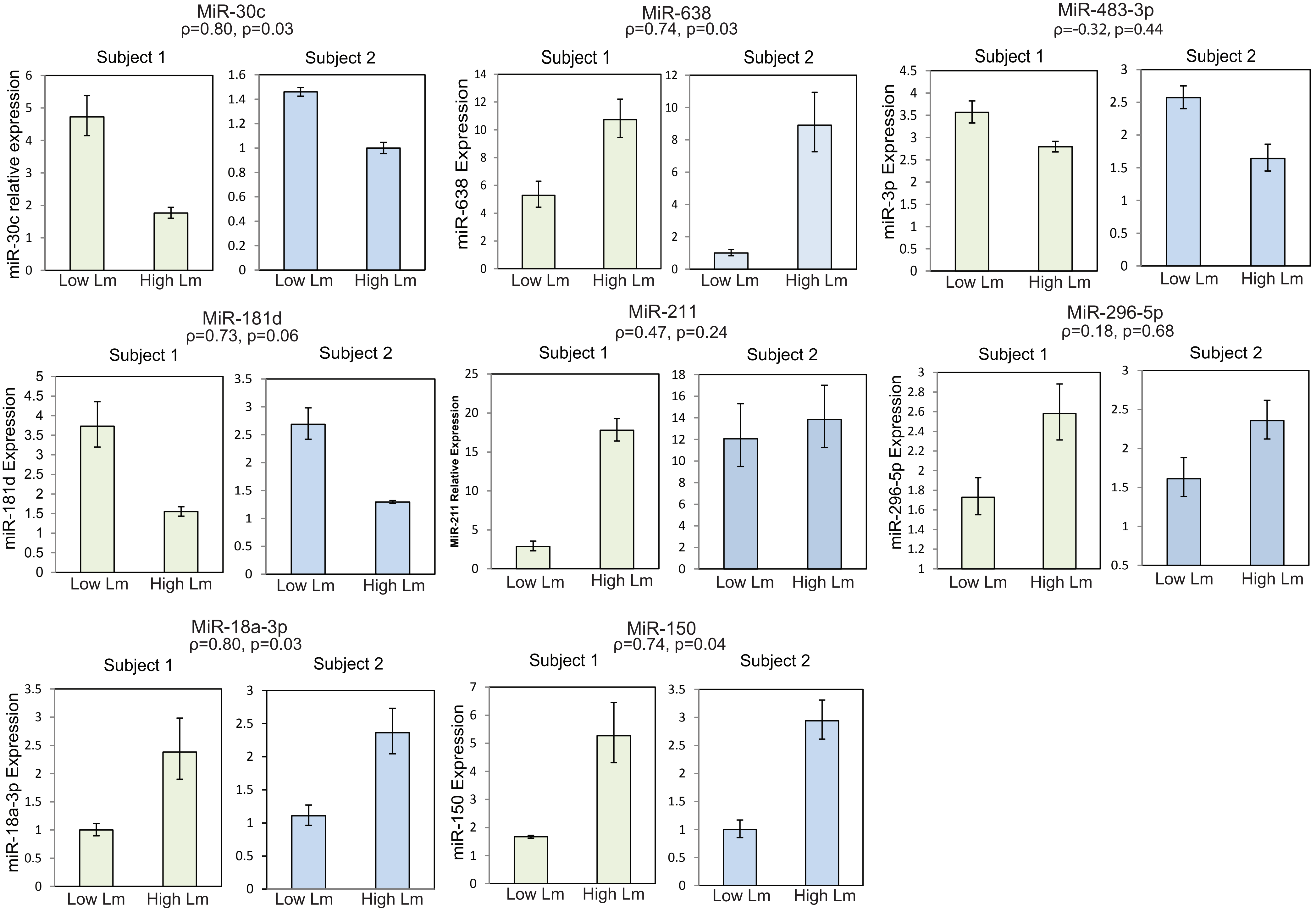
MiR-30c
ρ=0.80, p=0.03
Subject 1
Subject 2
miR-30c relative expression
Low Lm
High Lm
MiR-638
ρ=0.74, p=0.03
Subject 1
Subject 2
miR-638 Expression
Low Lm
High Lm
MiR-483-3p
ρ=-0.32, p=0.44
Subject 1
Subject 2
miR-3p Expression
Low Lm
High Lm
MiR-181d
ρ=0.73, p=0.06
Subject 1
Subject 2
miR-181d Expression
Low Lm
High Lm
MiR-211
ρ=0.47, p=0.24
Subject 1
Subject 2
MiR-211 Relative Expression
Low Lm
High Lm
MiR-296-5p
ρ=0.18, p=0.68
Subject 1
Subject 2
miR-296-5p Expression
Low Lm
High Lm
MiR-18a-3p
ρ=0.80, p=0.03
Subject 1
Subject 2
miR-18a-3p Expression
Low Lm
High Lm
MiR-150
ρ=0.74, p=0.04
Subject 1
Subject 2
miR-150 Expression
Low Lm
High Lm

Figure S2.

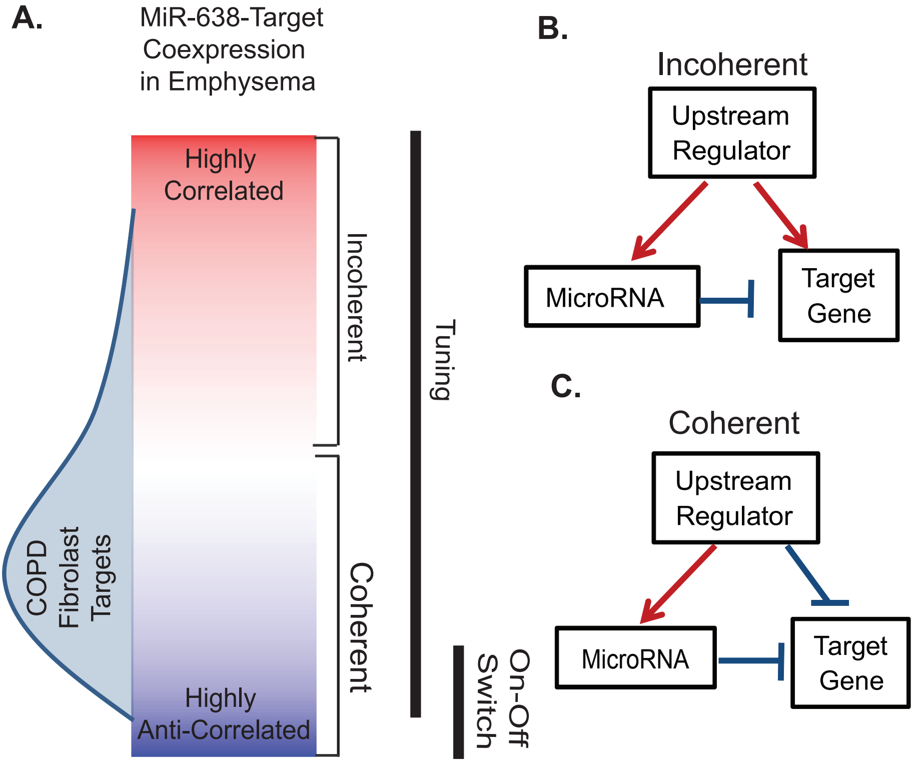
A.
MiR-638-Target
Coexpression
in Emphysema
Highly
Correlated
Highly
Anti-Correlated
COPD
Fibrolast
Targets
Incoherent
Coherent
Tuning
On-Off
Switch
B.
Incoherent
Upstream
Regulator
MicroRNA
Target
Gene
C.
Coherent
Upstream
Regulator
MicroRNA
Target
Gene

Figure S3.
